# Ambiguous apparent motion in exchanging disks

**DOI:** 10.1177/20416695241286023

**Published:** 2024-10-14

**Authors:** Stuart Anstis, Nihan Alp

**Affiliations:** Department of Psychology, University of California, San Diego, La Jolla, CA, USA; Department of Psychology, 52991 Sabanci Univeristy, Istanbul, Turkey

**Keywords:** illusion, motion perception, ambiguous

## Abstract

A large and a small disk with radii ***R*** and ***r***, side by side, exchange positions repetitively at 1.33 Hz. The motion is ambiguous. If ***r*/*R* **< 0.8 then observers perceive a single large disk jumping back and forth across a small static disk. But if ***r***/***R* **> 0.8, observers perceive two static disks that expand and contract in counter phase, with no motion across the gap between the disks. Observers respond to the movement of the *centroid*, which is greatest when ***r*/*R* **< 0.8.

Motion perception requires the integration of spatial and temporal elements. Apparent or stroboscopic motion is frequently used to study visual motion perception (reviewed by Rock, 1997). The perception of movement from stationary images shown in rapid succession, sheds light on how the visual system encodes motion (Yantis & Nakama, 1998). The brain uses spatial and temporal cues to interpret these rapid images as smooth motion (Whitney, 2002). There are two main motion perception systems. The position-based motion system relies on static cues such as similarity and proximity for integrating motion information, while the velocity-based motion system tends to disregard static cues (Duymaz & Alp, 2023).

We presented the stroboscopic motion of a large and a small disk that switched places. This motion was ambiguous (Movie 1). A recent study by Stepper et al. (2020) revealed that Ternus motion is influenced by perceived depth and size, suggesting that motion correspondence can occur at these processing levels. We found that when the size ratio ***r***/***R*** < 0.8, the larger disk seemed to jump back and forth, changing its position but maintaining its size. Conversely, when the disks were similar in size (***r***/***R*** > 0.8), they appeared to expand and contract in place. Our aim was to determine the size ratio at which these two percepts were equally likely.

**Movie 1.** Two disks of radii ***R*** and ***r*** repetitively exchange places at 1.33 Hz. (a) if the disks are very different in size (***r *** < *** *** 0.8 × ***R***) the display looks like a single large disk jumping back and forth. (b) If the disks are similar in size (***r *** > *** *** 0.8 × ***R***) then they look like two static disks expanding and contracting.

## Procedure

Movie 2 shows ten pairs of alternating black disks in which the radius of the smaller disk ***r*** ranged between 55% and 95% of the fixed radius ***R*** of the larger disk. The disks within each pair were positioned 4 × ***R*** apart. Disks were spaced out radially around a clockface, and their timings were given random phase shifts. The green numbers within the clockface (55% to 95%, give the relative size of the smaller disk (100 × ***r***/***R***). Students were never shown these numbers.

**Movie 2.** Ten pairs of alternating spots, with smaller spots of various sizes. Students picked the clock face number at which the spots transitioned from expansion /contraction to jumping.

Movie 3 shows examples of the four stimuli (the small disk radii ***r*** are arbitrarily set to 50% of the large disks ***R***, to encourage motion back and forth). Jumping and size change compete as alternative motion correspondences. We tried to bias the competition by weakening the jumping correspondence in two ways: Either by making the right-hand disks pale gray, reducing their Michelson contrast to 0.21 (row 2) or by doubling the inter-disk separation within each pair, from 4 × ***R*** to 8 × ***R*** (row 3). Movie 2 shows the “standard” version, but movies of all four conditions were shown to 43 psychology undergraduates at Sabanci University, Istanbul. Students recorded their perceptual transition points between expansion and back-and-forth motion as clockface numbers.

**Movie 3.** Transition points for different conditions ranged from 80% to 86.7%. High transition points means that jumping was more likely.

The results are shown in Movie 3. The transition point for the standard condition was 82.4%. Reducing the contrast of the right-hand disks (row 2) reduced the transition point insignificantly to 81.3%. Setting the two disks twice as far apart (row 3) reduced the transition point insignificantly to 80.1%. These modestly lower transition points meant that jumping was slightly less likely, and expansion/contraction was more likely. Adding an ISI had a strong effect in the opposite direction, supporting a velocity-based motion system that disregards static cues. It raised the transition point considerably to 86.7%, making back-and-forth motion more likely.

Why does adding an ISI make jumping more likely? Movie 4 shows three conditions: standard and ISI (from rows 1 and 4 of Movie 3), plus on the left an extra condition not shown to students, namely continuous size change. In all three movies the radius ***r*** of the smaller disk changes gradually over time from 50% to 90% of the radius ***R*** of the larger disk, and back again. This ratio ***r/R*** is displayed top left in Movie 4. Note that in the continuous movie, the sites of the two disks are never empty, so there is always evidence of stationary disks residing in both positions. Result: disks generally change sizes but not positions. In ISI on the other hand, the sites are empty for half the time, providing evidence that the disks do not always stay in the same places. Result: Jumping is often seen.

**Movie 4.** ***r*/*R*** increased steadily from 50% to 90% and back again (numbers on left). A smaller spot made jumping slightly more likely, but overall jumping was least likely for the continuous condition and most likely for the ISI condition.

## Rotary Motion

Movie 5a and b is a rotary version of the standard condition. The four rotating disks are the same in Movie 5a and b and the only difference is in the size of the stationary disks (***r*/*R* **= 0.166 in Movie 5a, 0.77 in Movie 5b). In Movie 5a, the four disks are always seen as rotating and it is impossible to see them as stationary disks changing in size. However, the disks in Movie 5b appear to pulsate in a place like a jelly, showing only an occasional hint of rotation.

**Movie 5.** Spots rotate clockwise in both rings. Differently-sized spots on the left (***r/R*** = 0.166) appear to rotate, but similar-sized spots on the right (***r/R*** = 0.77) just appear to pulsate in place.

## Centroids

We attribute the changeover between size change and jumping, to the *changing location of the centroid*. The centroid is defined as the average position of all the pixels in the movie. In Movie 6, the large disk of fixed radius ***R*** jumps back and forth, while the ***r/R*** of the small disk increases from 10% to 100% and back again. The red and blue vertical lines are the centroids of each movie frame. For small values of ***r*** (***r/R *** < 20%) the centroid hugs the large disk, but as the disks become more equal in size (***r/R *** > 80%) the centroids converge on a central position halfway between the two disks. Therefore, the centroids show that as the disks become more equal the net amount of motion decreases. Our results conform to perceptual principles of least action and of conservation of structural identity (Movie 7), as discussed by Farrell and Shepard (1981).

**Movie 6.** Moving centroid.

**Movie 7.** Triple ambiguity. Spots move horizontally, or vertically, or simply change size (after Gengerelli, 1948).
